# Supplementary material for: Influenza A virus ribonucleoproteins form liquid organelles at endoplasmic reticulum exit sites
Source: Nat Commun. 2019 Apr 9;10:1629. doi: 10.1038/s41467-019-09549-4 (PMC6456594; doi:10.1038/s41467-019-09549-4)
Supplement: Supplementary file 1 — Supplementary Information [file 41467_2019_9549_MOESM1_ESM.pdf]

SUPPLEMENTARY INFORMATION:

NCOMMS-18-24184A-Z

Alenquer M et al (2019)

INFLUENZA A VIRUS RIBONUCLEOPROTEINS FORM LIQUID ORGANELLES AT ENDOPLASMIC RETICULUM EXIT SITES

This section contains Supplementary Figures 1 – 7 and their respective legends, as well as Supplementary Methods.

SUPPLEMENTARY FIGURES:

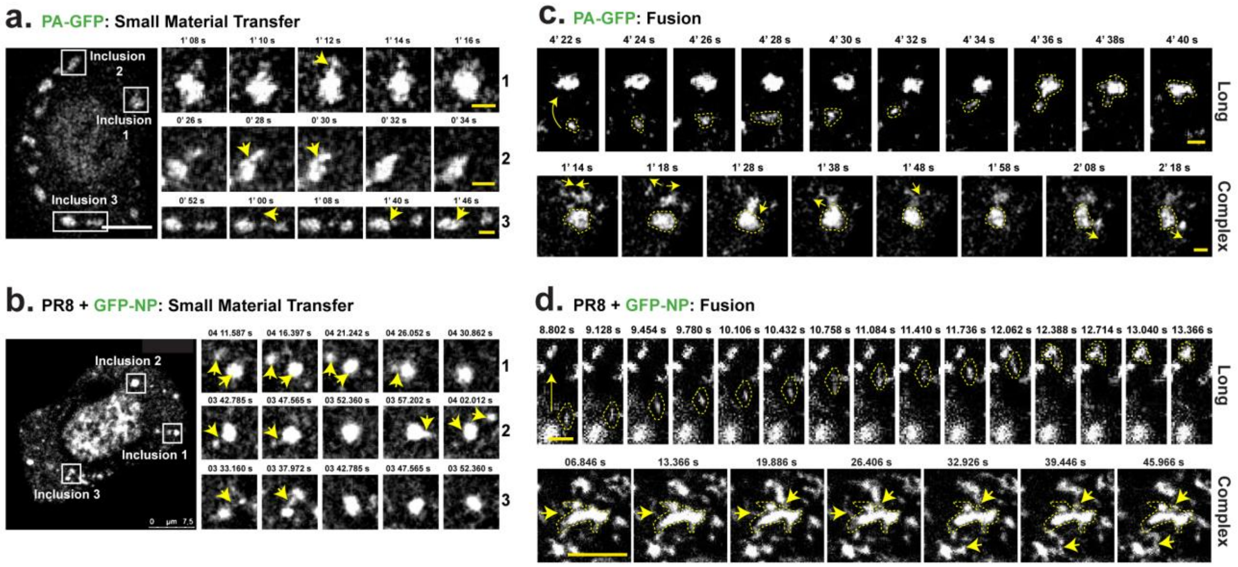

Supplementary Figure 1. Viral inclusions exchange material dynamically.

A549 cells were infected, at an MOI of 10, with PR8 PA-GFP virus for 16h (**a.**, **c.**). Alternatively, A549 cells were transfected with a plasmid encoding GFP-NP and co-infected with PR8 WT virus, at an MOI of 10, for 16 h (**b.**, **d.**). Cells were imaged under time-lapse conditions. **a., b.** A representative infected cell is shown in the large image, with selected inclusions marked by white boxes. Individual frames with single moving particles, from each inclusion, highlighted with yellow arrows are shown in the small panels. White Bar = 10 μm (**a.**) or 7.5 μm (**b.**). Yellow bar = 2 μm. Images were extracted from Supplementary Movie 4 (**a.**) or from Supplementary Movie 24 (**b.**). **c., d.** Individual frames show two distinct fusion events: complex short distance and at long distance. Yellow arrows highlight fusion or fission movements, whereas yellow dashed lines indicate the shape of inclusions. Images were extracted from Supplementary Movies 23 (**c.**) and 25 (**d.**). Bar = 2 μm.

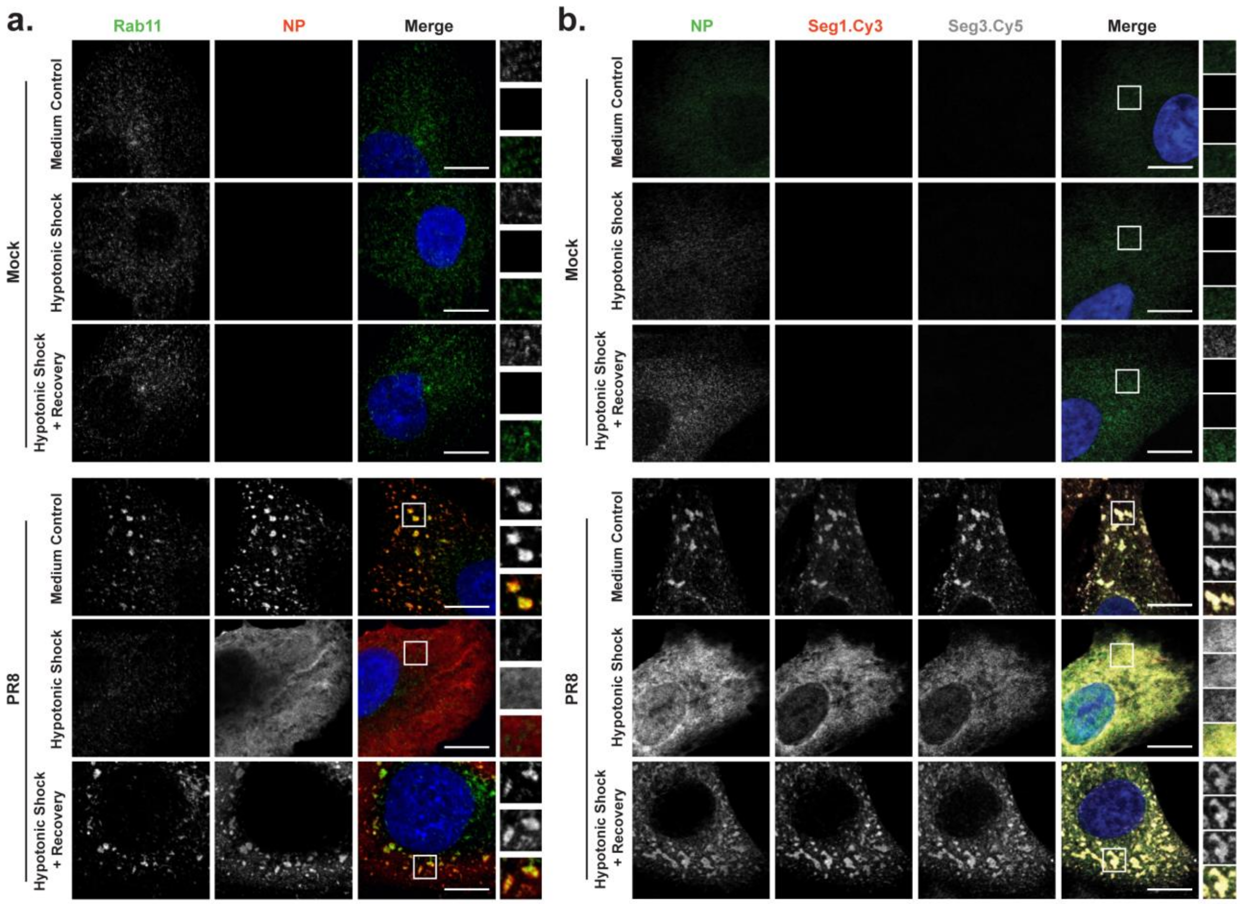

Supplementary Figure 2. Viral inclusions exhibit liquid-phase properties, dissolving upon a hypotonic shock.

A549 cells were mock-infected or infected, at an MOI of 3, with PR8 WT virus (**a.**, **b.**). At 16h post-infection, cells were treated with complete medium or water (80% water : 20% medium) for 10 min. Upon this period, cells were either fixed or allowed to recover for 1h in the presence of fresh complete medium. **a.** Cells were stained using antibodies against Rab11 (green) and viral NP (red). **b.** Cells were stained for viral NP (green) using an antibody and for viral RNA using probes against segment 1 (red) and 3 (gray). **a., b.** Areas highlighted by the white box are shown on the right of each panel. Bar = 10 μm. A single experiment representative of two independent experiments is shown.

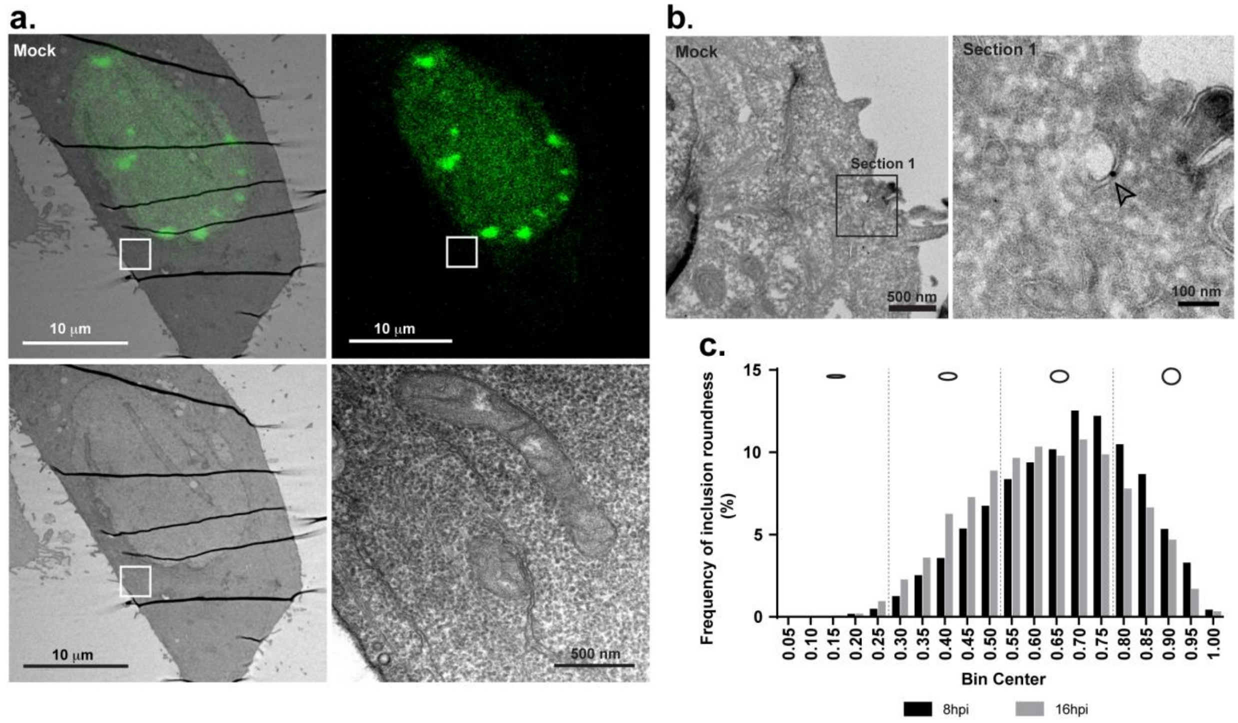

Supplementary Figure 3. Viral inclusions display a rounded shape that increases throughout infection.

a. HeLa cells were transfected with a plasmid encoding GFP-NP and mock-infected for 16h. Cells were imaged by confocal and electron microscopy and the resultant images were superimposed. A representative area of a mock-cell cytoplasm is highlighted by the white box and shown in greater detail. **b.** GFP-Rab11 WT cells were mock-infected for 16 h. Cells were stained for GFP (18 nm gold particles) and viral NP (6 nm gold particles). A representative section of a mock cell cytoplasm is highlighted by the black box and shown in greater detail (section 1). The arrowhead marks a Rab11 vesicle. No staining for NP was detected in mock-infected cells. **c.** A549 cells were infected, at an MOI of 3, with PR8 virus. Cells were fixed at the indicated times and stained for NP. The roundness of NP inclusions at each time of infection was calculated using the Shape Descriptor tool (Image J, NIH) and was plotted as the percentage relative frequency. The maximum value of roundness (1) corresponds to a circular structure, whereas the minimum value represents a linear structure (0). An average of 15 cells was analyzed per condition. A single experiment representative of two independent experiments is shown.

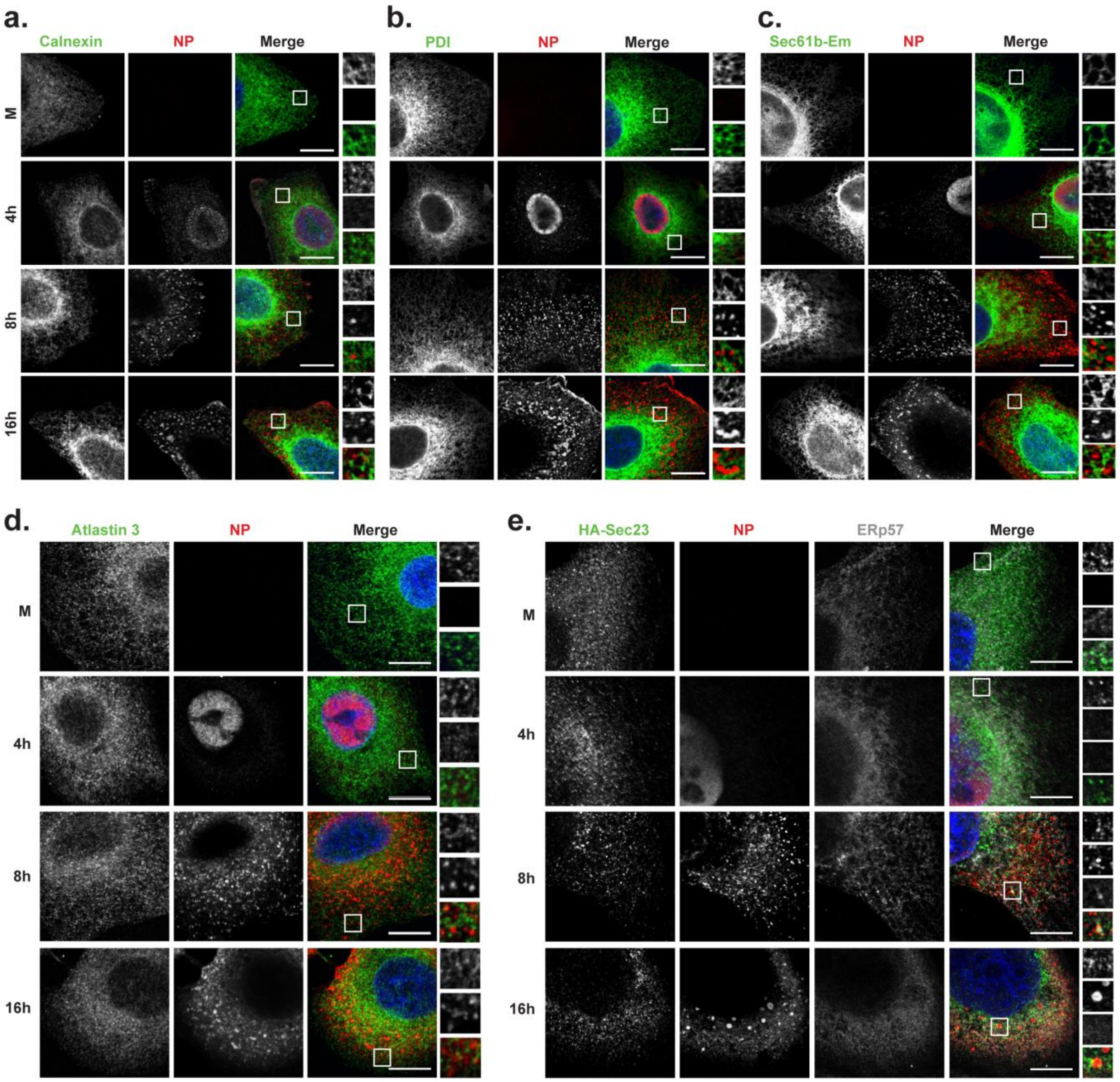

Supplementary Figure 4. Viral inclusions associate with ER exit sites.

A549 (**a.**, **b.**, **d.**) or Sec61β-Emerald (**c.**) cells were infected or mock-infected (M) with PR8 virus, at an MOI of 3, and fixed at the indicated times. **a.**, **b.** A549 cells were stained for the ER proteins (in green) calnexin (**a.**) or PDI (**b.**), and the viral NP protein (in red). **c.** Sec61β-Emerald cells were only stained for viral NP (in red). **d.** A549 cells were stained for the ER protein atlastin 3 (in green) and the viral NP protein (in red). **e.** HeLa cells were transfected with HA-Sec23 plasmid and, 24 h later, infected or mock-infected (M) with PR8 virus at an MOI of 10. Cells were fixed at the indicated times and stained for HA (green), viral NP (red) and host ERp57 (gray). **a–e.** Areas highlighted by the white box are shown on the

right of each panel. Bar = 10 μm. A single experiment representative of two independent experiments is shown.

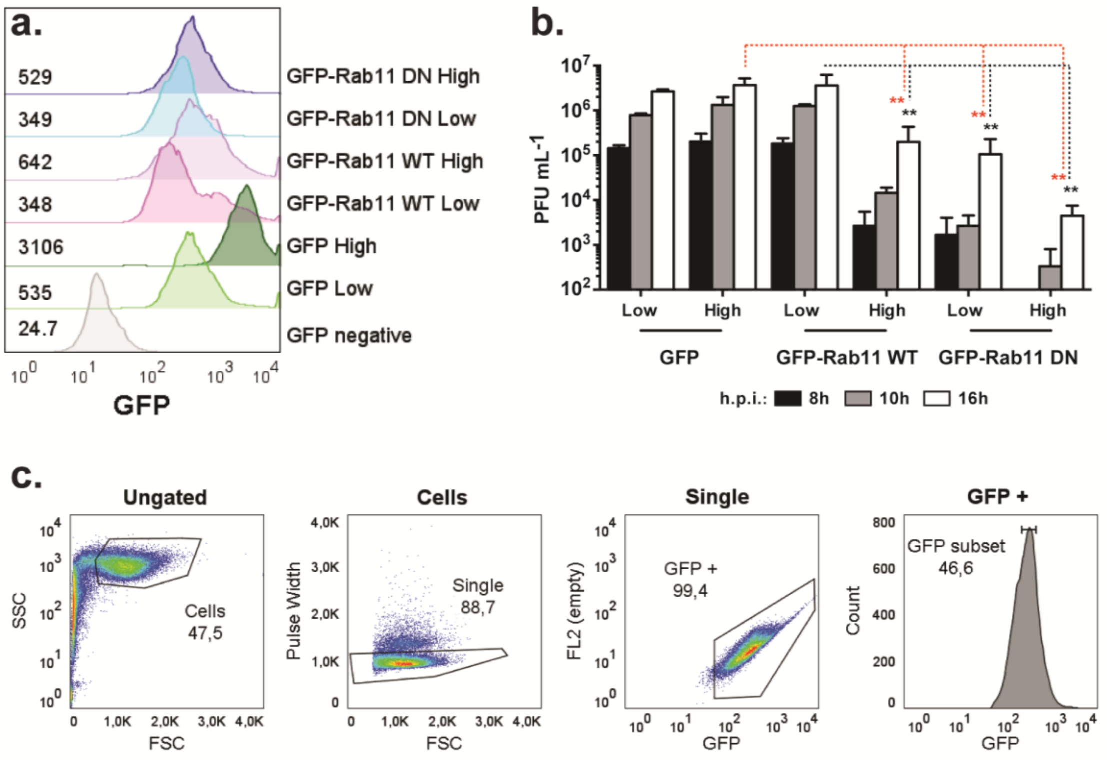

Supplementary Figure 5. Optimal viral production requires a functional Rab11.

A549 cells stably expressing GFP, GFP-Rab11 WT or GFP-Rab11 DN in low or high quantities (**a.**) were infected or mock-infected with PR8 WT virus, at an MOI of 3, for the indicated times (**b.**). **b.** Viral titres produced by each cell line were quantified and plotted as the mean ± s.e.m. of two independent experiments. Statistical analysis was performed using a Two-way ANOVA, followed by a Tukey multiple comparisons test (**p < 0.01). **c.** Gating strategy to define the cell populations presented in **a.**

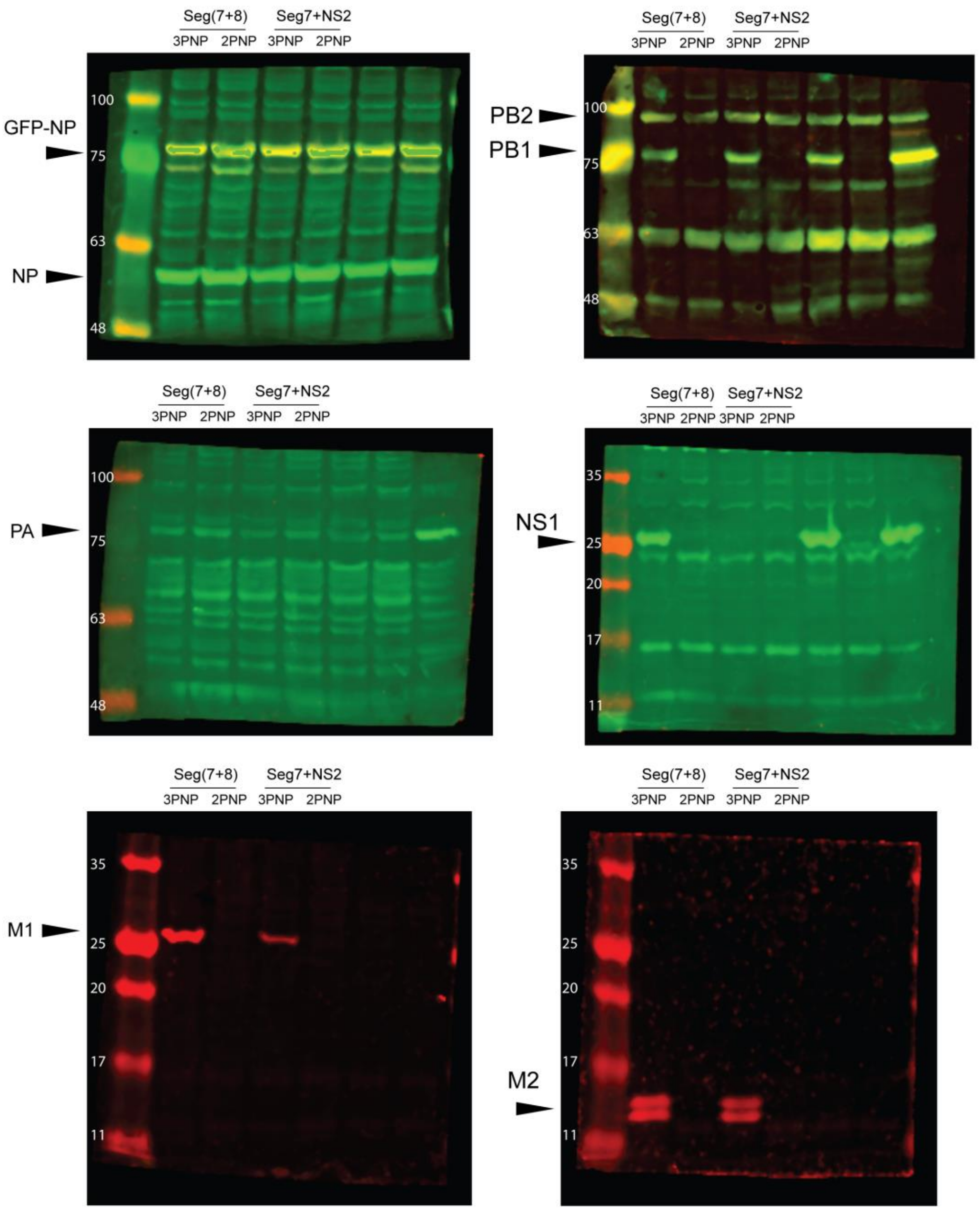

Supplementary Figure 6. Original western blots corresponding to Figure 1b.

The image depicts the original western blots from Figure 1b (main text), including the molecular weight markers (white numbers) and the detected proteins (black arrowheads). Western blotting was performed as described in Methods section (main text).

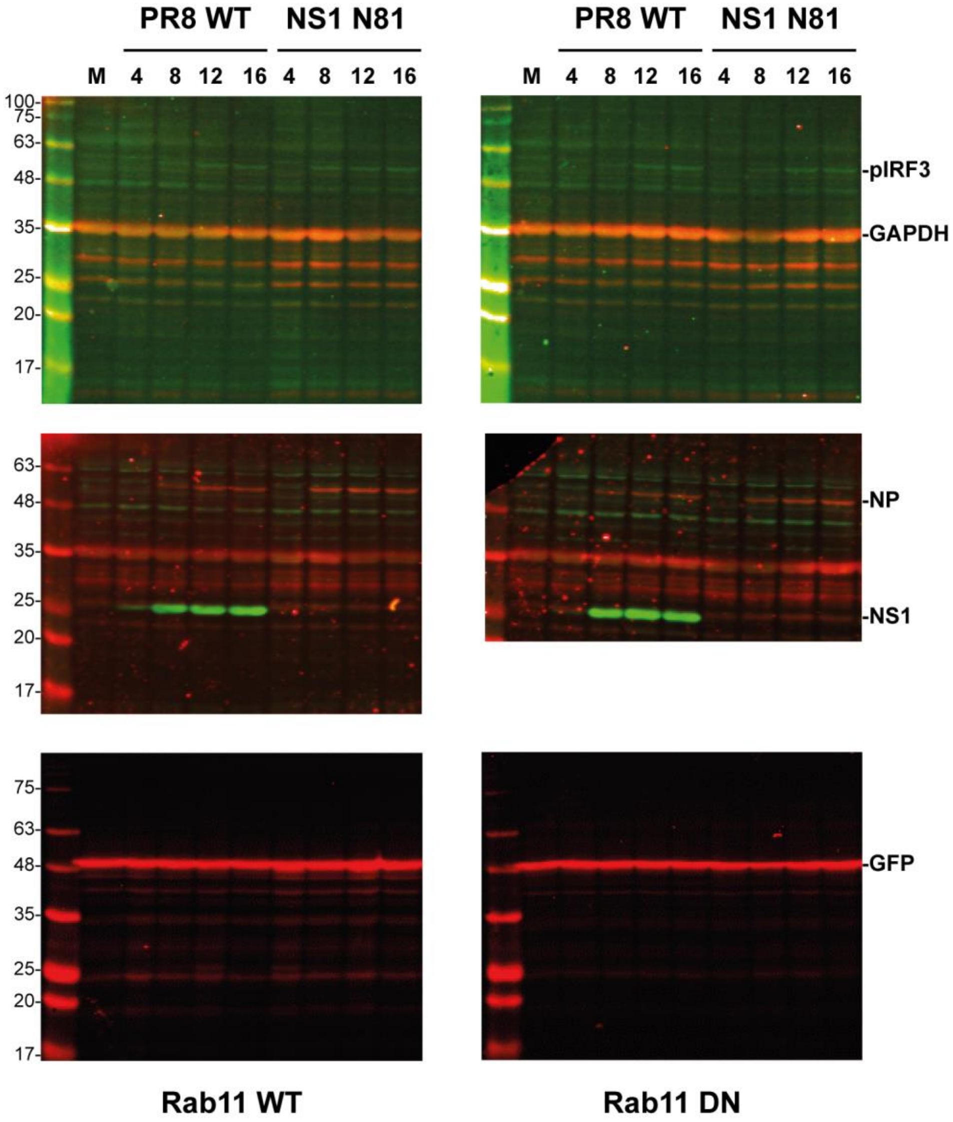

Supplementary Figure 7. Original western blots corresponding to Figure 8d.

The image depicts the original western blots from Figure 8d (main text), including the molecular weight markers (black numbers) and the detected proteins. Western blotting was performed as described in Methods section (main text).

SUPPLEMENTARY METHODS:

Cell sorting. A549 cells expressing GFP, GFP-Rab11 WT or GFP-Rab11 DN were grown to 90% confluency. A549 cells were prepared for cell sorting by detaching from the flasks with trypsin, followed by resuspension in PBS containing 2% FBS. Fluorescent cell sorting was carried out in a MoFlo (Beckman Coulter, Fort Collins, USA) with a 488 nm laser (200 mW air-cooled Sapphire, Coherent) at 140 mW for GFP excitation and detected using a 520/40 nm bandpass filter. Sorted cells were collected in eppendorfs containing culture media supplemented with 2.5ug/ml fungizone (Gibco) and 50 µg/ml Gentamicin (Gibco).
