# Supplementary material for: Influenza A virus ribonucleoproteins form liquid organelles at endoplasmic reticulum exit sites
Source: Nat Commun. 2019 Apr 9;10:1629. doi: 10.1038/s41467-019-09549-4 (PMC6456594; doi:10.1038/s41467-019-09549-4)
Supplement: Supplementary file 3 — Description of Additional Supplementary Files [file 41467_2019_9549_MOESM3_ESM.docx]

**Description of Additional Supplementary Files**

**File Name: Supplementary Movie 1**

**Description:** A549 cells were transfected with a plasmid encoding mCherry-NP and co-infected with PR8 PA-GFP virus, at an MOI of 10. At 16 hpi, a representative cell was imaged under timelapse conditions (every 2.0 s), for a maximum period of 5 min. An example of co-localisation between mCherry-NP and PA-GFP signal is highlighted by a white box. Bar = 10 μm.

**File Name: Supplementary Movie 2**

**Description:** A549 cells were infected with PR8 PA-GFP virus, at an MOI of 10. At 16 hpi, a representative cell was imaged under time-lapse conditions (every 2.0 s), for a maximum period of 5 min. Fission (0’ 02 s – 0’ 12 s) and fusion (0’ 52 s – 1’ 02 s) events are highlighted by a white box. Bar = 10 μm.

**File Name: Supplementary Movie 3**

**Description:** A549 cells were transfected with a plasmid encoding GFP-NP and co-infected with PR8 virus, at an MOI of 5. At 16 hpi, a representative cell was imaged under time-lapse conditions (every 4.8 s), during 01’40.938 s, displayed here from 04’59.812 to 05’03.013 s. Yellow squares highlight two distinct movements: short-distance fusion (05’12.651 – 05’41.453 s) and deformation/fission (06’05.496 – 06’34-343 s). Bar = 5 μm.

**File Name: Supplementary Movie 4**

**Description:** A549 cells were infected with PR8 PA-GFP virus, at an MOI of 10. At 4hpi, cells were treated with DMSO. At 16 hpi, a representative cell was imaged under time-lapse conditions (every 2.0 s), for a maximum period of 5 min. Examples of fusion and fission events are both highlighted by the white box at the indicated times. Bar = 10 μm.

**File Name: Supplementary Movie 5**

**Description:** A549 cells were infected with PR8 PA-GFP virus, at an MOI of 10. At 4hpi, cells were treated with Nocodazole (10 μg/mL). At 16 hpi, a representative cell was imaged under timelapse conditions (every 2.0 s), for a maximum period of 5 min. The white box indicates that viral inclusions have decreased movement caused by treatment with Nocodazole. Bar = 10 μm.

**File Name: Supplementary Movie 6**

**Description:** A549 cells were infected with PR8 PA-GFP virus, at an MOI of 10. At 4hpi, cells were treated with Latrunculin A (1 μM). At 16 hpi, a representative cell was imaged under time-lapse conditions (every 2.0 s), for a maximum period of 5 min. The white box shows fusion and fission movements in the presence of LatrunculinA. Bar = 10 μm.

**File Name: Supplementary Movie 7**

**Description:** A549 cells were transfected with a plasmid encoding GFP-NP and co-infected with PR8 WT virus, at an MOI of 10. At 8hpi, cells were treated with DMSO. At 10 hpi, a representative cell was imaged under time-lapse conditions (every 2.0 s), for a maximum period of 5 min. Examples of fusion and fission events are both highlighted by the white box at the indicated times. Bar = 10 μm.

**File Name: Supplementary Movie 8**

**Description:** A549 cells were transfected with a plasmid encoding GFP-NP and co-infected with PR8 WT virus, at an MOI of 10. At 8hpi, cells were treated with Nocodazole (10 μg/mL). At 10 hpi, a representative cell was imaged under time-lapse conditions (every 2.0 s), for a maximum period 5 of 5 min. The white box indicates that viral inclusions have decreased movement caused by treatment with Nocodazole. Bar = 10 μm.

**File Name: Supplementary Movie 9**

**Description:** A549 cells were transfected with a plasmid encoding GFP-NP and co-infected with PR8 WT virus, at an MOI of 10. At 8hpi, cells were treated with Latrunculin A (1 μM). At 10 hpi, a representative cell was imaged under time-lapse conditions (every 2.0 s), for a maximum period of 5 min. The white box shows fusion and fission movements in the presence of LatrunculinA. Bar = 10 μm.

**File Name: Supplementary Movie 10**

**Description:** A549 cells were transfected with a plasmid encoding GFP-NP and co-infected with PR8 virus, at an MOI of 5. At 16 hpi, a representative cell was imaged under time-lapse conditions (every 1.44 s), for a maximum period of 09’58.458 s. The addition of regular growth medium occurred at 1’33.505 s. Bar = 7.5 μm.

**File Name: Supplementary Movie 11**

**Description:** A549 cells were transfected with a plasmid encoding GFP-NP and co-infected with PR8 virus, at an MOI of 5. At 16 hpi, a representative cell was imaged under time-lapse conditions (every 1.44 s), for a maximum period of 15’06.336 s. The addition of water (hypotonic shock) occurred at 1’03.328 s. Bar = 10 μm.

**File Name: Supplementary Movie 12a & 12b**

**Description:** A549 cells were transfected with a plasmid encoding GFP-NP and co-infected with PR8 virus, at an MOI of 10. At 8 hpi, a representative cell was imaged under time-lapse conditions (every 2.0 s). 12a. Before addition of 5% 1,6-Hexanediol (imaged for 1 min). 12b. After addition of 5% 1,6-Hexanediol (recorded for a maximum period of 13’44s). White box highlights an area of the cytoplasm where viral inclusions can be seen to dissolve. Bar = 10 μm.

**File Name: Supplementary Movie 13a & 13b**

**Description:** A549 cells were infected with PR8 PA-GFP virus, at an MOI of 10. At 16 hpi, a representative cell was imaged under time-lapse conditions (every 2.0 s). 13a. Before addition of regular media (imaged for 1 min). 13b. After addition of regular media (recorded for a maximum period of 10 min). The large white box highlights an area of the cytoplasm where viral inclusions maintain their movements. Smaller white boxes show 3 distinct inclusions exchanging small material amounts. Bar = 10 μm.

**File Name: Supplementary Movie 14a & 14b**

**Description:** A549 cells were infected with PR8 PA-GFP virus, at an MOI of 10. At 16 hpi, a representative cell was imaged under time-lapse conditions (every 2.0 s). 14a. Before addition of water (hypotonic shock, imaged for 1 min). 14b. After addition of water (recorded for a maximum period of 10 min). White box highlights an area of the cytoplasm where viral inclusions can be seen to dissolve. Bar = 10 μm.

**File Name: Supplementary Movie 15a & 15b**

**Description:** A549 cells were infected with PR8 PA-GFP virus, at an MOI of 10. At 16 hpi, a representative cell was imaged under time-lapse conditions (every 2.0 s). 15a. Before addition of 5% 1,6-Hexanediol (imaged for 1 min). 15b. After addition of 5% 1,6-Hexanediol (recorded for a maximum period of 10 min). White box highlights an area of the cytoplasm where viral inclusions can be seen to dissolve. Bar = 10 μm.

**File Name: Supplementary Movie 16**

**Description:** A549 cells were transfected with a plasmid encoding GFP-NP and co-infected with PR8 WT virus, at an MOI of 5. At 16 hpi, a representative cell was imaged under time-lapse conditions (every 0.326 s), for a maximum period of 66.830 s. Two areas of viral NP clusters, highlighted in purple and cyan boxes, were photobleached at 1.630 s. The photobleached regions are marked by a yellow circle. Bar = 10 μm.

**File Name: Supplementary Movie 17**

**Description:** Sec61β-Emerald cells were transfected with mCherry-NP and infected with PR8 WT virus, at an MOI of 10, for 12 h. A representative cell was imaged under time-lapse conditions (every 4.8 s), for a maximum period of 05’58.363 s. Yellow rectangle highlights the movements of the ER (green) and viral NP clusters (red). Bar = 2.5 μm.

**File Name: Supplementary Movie 18**

**Description:** A549 cells were co-transfected with plasmids encoding mCherry-NP and ER-GFP and infected with PR8 WT virus, at an MOI of 10, for 12h. A representative cell was imaged under time-lapse conditions (every 4.8s), for a maximum period of 02’29.642 s. Yellow box highlights the movements of the ER (green) and viral NP clusters (red). Bar = 2.5 μm.

**File Name: Supplementary Movie 19**

**Description:** A549 cells were co-transfected with plasmids encoding mCherry-NP and GFP-Sec16 and mock-infected (Mock) for 16 h. A representative cell was imaged under time-lapse conditions (every 4.8 s), during 97.287s, displayed here from 04’03.391 to 05’00.678 s. Yellow box highlights movements of Sec16 (green) and viral NP clusters (red). Bar = 7.5 μm.

**File Name: Supplementary Movie 20**

**Description:** A549 cells were co-transfected with plasmids encoding mCherry-NP and GFP-Sec16 and with PR8 WT virus for 16 h. A representative cell was imaged under time-lapse conditions (every 3.95 s), for a maximum period of 03’03.550 s. Yellow boxes highlights two examples (16h.1 and 16h.2) of movements of Sec16 (green) and viral NP clusters (red). Bar = 7.5 μm.

**File Name: Supplementary Movie 21**

**Description:** A549 cells were co-transfected with plasmids encoding GFP-NP and co-infected with PR8 WT virus for 10-16h. A representative cell was imaged under time-lapse conditions (every 4.8 s), for a maximum period of 3’45 s. The addition of regular media occurred right at the start of imaging. Bar = 10 μm.

**File Name: Supplementary Movie 22**

**Description:** A549 cells were co-transfected with plasmids encoding GFP-NP and co-infected with PR8 WT virus for 10-16h. A representative cell was imaged under time-lapse conditions (every 4.8 s), for a maximum period of 13’55 s. The addition of BFA (2 μg/mL) occurred at 01’45s. Bar = 10 μm.

**File Name: Supplementary Movie 23**

**Description:** A549 cells were infected with PR8 PA-GFP virus, at an MOI of 10. At 16 hpi, a representative cell was imaged under time-lapse conditions (every 2.0 s), for a maximum period of 5 min. The white boxes highlight long distance (4’ 22 s – 4’ 40 s) and complex (1’ 14 s – 2’ 18 s) fusion movements. Bar = 10 μm.

**File Name: Supplementary Movie 24**

**Description:** A549 cells were transfected with a plasmid encoding GFP-NP and co-infected with PR8 WT virus, at an MOI of 5. At 16 hpi, a representative cell was imaged under time-lapse conditions (every 4.8s), during 01’41.067 s, displayed here from 03’18.745 to 04’59.812 s. Three areas of viral NP clusters are highlighted in yellow boxes. Bar = 7.5 μm.

**File Name: Supplementary Movie 25**

**Description:** A549 cells were transfected with a plasmid encoding GFP-NP and co-infected with PR8 WT virus, at an MOI of 5. At 16 hpi, a representative cell was imaged under time-lapse conditions (every 0.326 s), for a maximum period of 69.112s. Yellow squares highlight two distinct movements: complex transfer (6.846 – 46.292s) and long distance fusion (8.802 – 13.366s). Bar = 10 μm.
