# Supplementary material for: Influenza A virus ribonucleoproteins form liquid organelles at endoplasmic reticulum exit sites
Source: Nat Commun. 2019 Apr 9;10:1629. doi: 10.1038/s41467-019-09549-4 (PMC6456594; doi:10.1038/s41467-019-09549-4)
Supplement: Supplementary file 33 — Reporting Summary [file 41467_2019_9549_MOESM33_ESM.pdf]

natureresearch

Corresponding author(s): Maria João Amorim

Last updated by author(s): Mar 11, 2019

Reporting Summary

Nature Research wishes to improve the reproducibility of the work that we publish. This form provides structure for consistency and transparency in reporting. For further information on Nature Research policies, see Authors & Referees and the Editorial Policy Checklist.

Statistics

For all statistical analyses, confirm that the following items are present in the figure legend, table legend, main text, or Methods section.

n/a	Confirmed	
☐	☒	The exact sample size (*n*) for each experimental group/condition, given as a discrete number and unit of measurement
☐	☒	A statement on whether measurements were taken from distinct samples or whether the same sample was measured repeatedly
☐	☒	The statistical test(s) used AND whether they are one- or two-sided<br>*Only common tests should be described solely by name; describe more complex techniques in the Methods section.*
☒	☐	A description of all covariates tested
☐	☒	A description of any assumptions or corrections, such as tests of normality and adjustment for multiple comparisons
☐	☒	A full description of the statistical parameters including central tendency (e.g. means) or other basic estimates (e.g. regression coefficient) AND variation (e.g. standard deviation) or associated estimates of uncertainty (e.g. confidence intervals)
☐	☒	For null hypothesis testing, the test statistic (e.g. *F*, *t*, *r*) with confidence intervals, effect sizes, degrees of freedom and *P* value noted<br>*Give P values as exact values whenever suitable.*
☒	☐	For Bayesian analysis, information on the choice of priors and Markov chain Monte Carlo settings
☒	☐	For hierarchical and complex designs, identification of the appropriate level for tests and full reporting of outcomes
☐	☒	Estimates of effect sizes (e.g. Cohen's *d*, Pearson's *r*), indicating how they were calculated

Our web collection on statistics for biologists contains articles on many of the points above.

Software and code

Policy information about availability of computer code

Data collection	No software was used
Data analysis	ImageJ (NIH) v1.49b

For manuscripts utilizing custom algorithms or software that are central to the research but not yet described in published literature, software must be made available to editors/reviewers. We strongly encourage code deposition in a community repository (e.g. GitHub). See the Nature Research guidelines for submitting code & software for further information.

Data

Policy information about availability of data

All manuscripts must include a data availability statement. This statement should provide the following information, where applicable:

- Accession codes, unique identifiers, or web links for publicly available datasets
- A list of figures that have associated raw data
- A description of any restrictions on data availability

The authors declare that the data supporting the findings of this study are available within the paper and its Supplementary Information files

Field-specific reporting

Please select the one below that is the best fit for your research. If you are not sure, read the appropriate sections before making your selection.

☒ Life sciences ☐ Behavioural & social sciences ☐ Ecological, evolutionary & environmental sciences

For a reference copy of the document with all sections, see nature.com/documents/nr-reporting-summary-flat.pdf

Life sciences study design

All studies must disclose on these points even when the disclosure is negative.

Sample size	Our choice of sample size was based on the background variations of the parameters being analyzed. For high variation we opted for high numbers (up to 100 cells analyzed) and for parameters that did not vary much we used 30 cells. This was based on knowledge from previous experiments.
Data exclusions	No data was excluded from this study. We think that outliers, if present, are important biological parameters of the analysis.
Replication	The experimental findings were repeated at least twice, but the majority was repeated three times. In all cases, the result or tendency was verified and biological significance confimed using statistical analysis tests (stated in the manuscript)
Randomization	Not relevant to our study.
Blinding	Blinding was not performed. However, experiments were independently confirmed and repeated by several of the co-authors in this manuscript. With this strategy, we are confident with all the data presented.

Reporting for specific materials, systems and methods

We require information from authors about some types of materials, experimental systems and methods used in many studies. Here, indicate whether each material, system or method listed is relevant to your study. If you are not sure if a list item applies to your research, read the appropriate section before selecting a response.

Materials & experimental systems

n/a	Involved in the study	
☐	☒	Antibodies
☐	☒	Eukaryotic cell lines
☒	☐	Palaeontology
☒	☐	Animals and other organisms
☒	☐	Human research participants
☒	☐	Clinical data

Methods

n/a	Involved in the study	
☒	☐	ChIP-seq
☐	☒	Flow cytometry
☒	☐	MRI-based neuroimaging

Antibodies

Antibodies used	For immunofluorescence - Rabbit polyclonal against: Rab11a (1:100; Life Technologies, 715300), HA tag (1:500; Abcam, 9110), calnexin (1:1000, Abcam, 22595), atlastin 3 (1:100; Proteintech, 16921-1-AP) and NP (1:1000; gift from Prof Paul Digard); Mouse monoclonal against: NP (1:1000; Abcam, 20343), virus HA (neat; gift from Prof Paul Digard), M2 (1:500, Abcam, 5416), PDI (1:500, Life Technologies, MA3-019) and Sec31A (1:100; BD Biosciences, 612350); Goat polyclonal against ERp57 (1:200; Sicgen, AB0003-200). Secondary antibodies were all from the Alexa Fluor range (1:1000; Life Technologies).<br><br>For western blotting - Rabbit polyclonal against: pIRF3 (1:1000; Cell Signal, 4947), virus NP (1:1000), PB1, PB2, PA and NS1 (all at 1:500), kindly provided by Prof. Paul Digard, Roslin Institute, UK; Goat polyclonal against: green fluorescent protein (GFP) (1:2000; Sicgen, AB0020), GAPDH (1:2000; Sicgen, AB0049) and virus M1 (1:500; Abcam, 20910); Mouse polyclonal against: virus M2 (1:500; Abcam, 5416). The secondary antibodies used were from IRDye range (1:10000; LI-COR Biosciences).
Validation	Data provided in the manuscript is sufficient to validate primary antibodies used in this study. All commercial antibodies used have been validated, as can be checked at the manufacturer's bulletins. All homemade or offered antibodies have been validated previously using siRNA depletion for host factors, or in case of viral proteins using mock infected cells and mutants. These validations were published before in [Vale-Costa et al (2016) Journal of Cell Science 129, 1697-1710; Amorim et al (2011) J Virology, 85, 4143-4156].

Eukaryotic cell lines

Policy information about cell lines

Cell line source(s)	MDCK, HEK 293T, HeLa and A549 (Paul Digard, Roslin Institute, UK); HeLa Sec61b-Emerald (Christophe Dehio, Biozentrum, University of Basel, Switzerland); A549 GFP-Rab11 WT and DN were developed by us (Vale-Costa et al, 2016, Journal of Cell Science, 129, 1697-1710); A549 GFP, GFP-Rab11 WT and DN - High or Low expressers - were developed by us during this study. To characterize the latter, we performed several tests: 1) growth curve, 2) co-localisation with Rab11 using antibodies, 3) GFP expression levels by flow cytometry and finally 4) permissiveness to viral infection.
Authentication	We have not performed authentication following the guidelines of ICLAC.

Mycoplasma contamination	All cell lines used throughout this study were regularly tested for mycoplasma contamination with the LookOut Mycoplasma PCR Detection kit (Sigma, MP0035), using JumpStart Taq DNA Polymerase (Sigma, D9307)
Commonly misidentified lines (See ICLAC register)	HeLa cells were used in this study because they are transfectable to high efficiency and because they are suitable for microscopy. However, our study used several distinct cell lines to corroborate all the findings thus providing confidence in the results.

Flow Cytometry

Plots

Confirm that:

☒ The axis labels state the marker and fluorochrome used (e.g. CD4-FITC).

☒ The axis scales are clearly visible. Include numbers along axes only for bottom left plot of group (a 'group' is an analysis of identical markers).

☒ All plots are contour plots with outliers or pseudocolor plots.

☒ A numerical value for number of cells or percentage (with statistics) is provided.

Methodology

Sample preparation	A549 cells expressing either GFP, GFP-Rab11aWT and GFP-Rab11aDN were grown to 90% confluency. A549 cells were prepared for cell sorting by detaching from the flasks with trypsin, followed by resuspension in PBS containing 2% FBS
Instrument	MoFlo (Beckman Coulter, Fort Collins, USA) with a 488 nm laser (200 mW air-cooled Sapphire, Coherent) using a 520/40 nm bandpass filter
Software	Summit V4.3.01 Build 2449
Cell population abundance	NA
Gating strategy	Cells were sorted via SSC plotted against FSC, followed by pulse width plotted against FSC to gate singlets. Positive GFP cells were gated against cells expressing no GFP and a GFP subset was sorted into eppendorfs containing DMEM supplemented with 10% FBS, 2mM L-Glutamine, 1% Penicillin/Streptomycin, 2.5ug/ml Fungizone and 50ug/ml Gentamicin.

☒ Tick this box to confirm that a figure exemplifying the gating strategy is provided in the Supplementary Information.
